# Enhancement of Mixing Performance of Two-Layer Crossing Micromixer through Surrogate-Based Optimization

**DOI:** 10.3390/mi12020211

**Published:** 2021-02-19

**Authors:** Shakhawat Hossain, Nass Toufiq Tayeb, Farzana Islam, Mosab Kaseem, P.D.H. Bui, M.M.K. Bhuiya, Muhammad Aslam, Kwang-Yong Kim

**Affiliations:** 1 Department of Industrial and Production Engineering, Jashore University of Science and Technology, Jashore 7408, Bangladesh; 2 Gas Turbine Joint Research Team, University of Djelfa, Djelfa 17000, Algeria; toufiknaas@gmail.com; 3 Department of Nanotechnology and Advanced Materials Engineering, Sejong University, Seoul 05006, Korea; farzanaislam2003@gmail.com (F.I.); mosabkaseem@sejong.ac.kr (M.K.); 4 Department of Mechanical Engineering, University of Tulsa, Tulsa, OK 74104, USA; phuc-bui@utulsa.edu; 5 Department of Mechanical Engineering, Chittagong University of Engineering & Technology (CUET), Chittagong 4349, Bangladesh; mkamalcuet@gmail.com; 6 Department of Chemical Engineering, Lahore Campus, COMSATS University Islamabad (CUI), Lahore 53720, Pakistan; maslam@cuilahore.edu.pk; 7 Department of Mechanical Engineering, Inha University, 100 Inha-ro, Michuhol-gu, Incheon 22212, Korea

**Keywords:** Navier–Stokes equations, mixing index, passive micromixers, optimization, RBNN

## Abstract

Optimum configuration of a micromixer with two-layer crossing microstructure was performed using mixing analysis, surrogate modeling, along with an optimization algorithm. Mixing performance was used to determine the optimum designs at Reynolds number 40. A surrogate modeling method based on a radial basis neural network (RBNN) was used to approximate the value of the objective function. The optimization study was carried out with three design variables; viz., the ratio of the main channel thickness to the pitch length (H/PI), the ratio of the thickness of the diagonal channel to the pitch length (W/PI), and the ratio of the depth of the channel to the pitch length (d/PI). Through a primary parametric study, the design space was constrained. The design points surrounded by the design constraints were chosen using a well-known technique called Latin hypercube sampling (LHS). The optimal design confirmed a 32.0% enhancement of the mixing index as compared to the reference design.

## 1. Introduction

The recent advancements in miniaturized research in biochemistry and biomedicine demand development in the area of microfluidic systems. Micromixers are one of the fundamental components of micro total analysis systems (μ-TAS) and microfluidic systems [1,2]. A microchannel has geometrical dimensions typically in the order of a few microns. The flow inside such a microchannel is characterized by a small value of Reynolds numbers (Re), where the inertia forces of a fluid are much lower than the viscous forces. Micromixing is largely controlled by molecular diffusion, particularly at low Reynolds numbers. Most importantly, the fluid flow within the micro devices is a ubiquity of laminar.Apart from the various advantages of micro devices, such as the high surface-to-volume ratio, portability, and decreased analysis time, there are also challenges to achieving high mixing efficiency, as development of molecular diffusion is extremely sluggish [3].

On the basis of their working principles, micromixers have been classified into passive micromixers and active micromixers. Mixing performance is increased by employing external energy sources within an active micromixer. There are various methods available for flow manipulation to enhance mixing, such as pressure, acoustic, thermal, magnetic, electrokinetic, etc. In an active micromixer, higher mixing efficiency can be achieved in a very short mixing length. However, these micromixers require complex manufacturing methods, and their integration into a microfluidic system is also difficult. In a passive micromixer, the mixing is simply increased with the help of a modification in the geometrical structure of the microchannel. Recently, passive micromixers have been the superior preference for researchers over active micromixers because of their easier fabrication and their incorporation technique into microfluidic systems [4,5,6,7]. 

Commercial software using computational fluid dynamics (CFD) is one of the most consistent and popular tools for fluid flow structural analyses, as well as for evaluating the performance of microfluidic devices [8,9,10,11]. In recent times, to achieve efficient mixing, numerous micro devices have been proposed by researchers. In a laminar flow regime, the fluid flow is based on a chaotic mechanism that is stimulated by the cyclic disturbance of the flow, which can evidently progress the mixing performance [12,13]. The three-dimensional serpentine microchannel [14] was designed and fabricated to generate chaotic advection using the recurring behavior of stretching and folding phenomena of the fluid flow streams. However, the proposed micromixer can work well and produce chaotic advection with a reasonably higher Reynolds number (>25). A micromixer incorporated with oblique grooves at the bottom, proposed by Stroock et al. [15], could create three-dimensional twisting flows. The authors observed chaotic advection in the microchannel with herringbone grooves because of the sporadic velocity fields of the fluid streams. Chaotic microchannels with rectangular obstacles on the slanted grooves designed by Kim et al. [16], because of the regular disturbance of the flow streams over the slanted grooves, created a chaotic mixing mechanism and enhanced the performance of the device. Chaotic fluid flow mechanisms were observed by Wang and Yang [17] using an overlapping crisscross microchannel; the device could generate chaotic advections by enhancing and shrinking the streams of miscible fluid. A two-layer crossing microchannel (TLCCM) based on chaotic mixing behavior has been reported [18]; the study demonstrated that the micromixer showed high performance at Reynolds number (Re < 0.2). 

For performance development of the available micromixers, the design optimization technique using the CFD tool has progressively and increasingly become the method preferred by researchers. Structural optimization of a micromixer with a slanted groove microchannel was performed [11]; using electroosmotic flow, the study demonstrated that the performance of the devices was remarkably improved and that performance depended on the depth and angle of the pattern grooves. For augmentation of the performance of a herringbone-grooves microchannel, a detailed optimization study based on the radial basis neural network (RBNN) technique was used by Ansari and Kim [19] using three different parameters. Lynn and Dandy [20] performed structural optimization of a micromixer using four different parameters; the performance of the micromixer was improved surprisingly by the investigated parameters. Hosasin et al. [21] optimized a modified Tesla structure using a weighted-average (WTA) surrogate model with two different objective functions to originate a single-objective optimization problem. A micromixer with a herringbone grooves device was optimized [22,23] using two functions: pressure losses and mixing performance. A micromixer with sigma structure [24] and convergent-divergent sinusoidal walls [25] was optimized using a multi-objective optimization technique. Objective function values were correlated using the concave shape of a Pareto-optimal font. Performance of the asymmetrical shape with a split-and-recombined [26] microchannel was improved through an optimization technique at Re = 20. The authors performed both single-objective and multi-objective optimizations using particle swarm and genetic algorithm optimization methods. The study concluded that improvement of the mixing effectiveness (by 58.9%) was achieved with the reference design using the single-objective optimization technique. The multi-objective optimization showed a 48.5% improvement of the mixing index and a 55% decrease of the pressure drop with the reference device.

Through a literature survey, we have established that a numerical optimization process using three-dimensional CFD has been a constructive tool for improvement of existing micromixers. This study performed single-objective optimizations for the further enhancement of micromixer performance in terms of mixing index, as proposed by Ahmed et al. [27]. The optimization study was carried out with three dimensionless design variables at Re = 40. The design points surrounded by the design constraints were chosen using the well-known Latin hypercube sampling (LHS) technique. The surrogate modeling method based on the RBNN was applied to approximate the objective function. 

## 2. Design of the Proposed Microchannel

**Figure 1:**
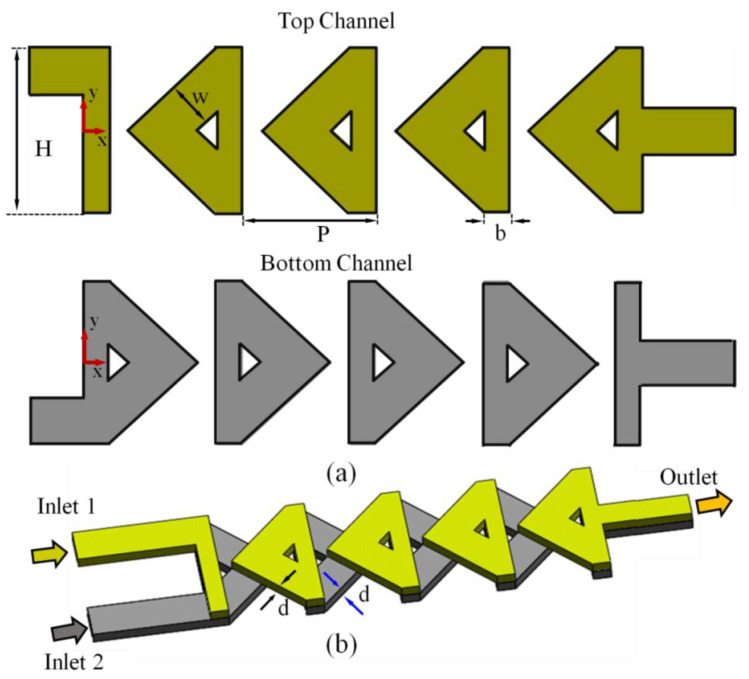
Two-layer crossing micromixer [27]. (**a**) Two-dimensional design of the top layer and bottom layer, respectively. (**b**) Proposed micromixer with three-dimensional image; both channels are interrelated at the center of the crossing-unit and the vertical sections.

In our previous study [27], to inspect the mixing performance a micromixer including a two-layer (top layer and bottom layer) microchannel was proposed. The two inlet channels were joined with the main crossing channel at an angle of 90°, as shown in Figure 1a. The fluids were interrelated all the way through the vertical segments and at the center of every crossing segment. A consecutive structure of ten mixing units split and reconnected the fluid streams in a cyclic approach. The geometric dimensions of the projected micromixer were as follows: diagonal channel width (W), pitch length (PI), thickness of the main channel (H), depth of the single channel (d), vertical segment (b), and number of mixing units were 0.15 mm, 0.64 mm, 1.07 mm, 0.15 mm, 0.3 mm, 0.15 mm, and 10, respectively. The dimensions of the inlet channel were 0.15 × 0.3 mm, and the outlet channel was 0.3 × 0.3 mm. 

## 3. Numerical Scheme 

The present section formulates the numerical model used in this study for the proposed design of the microchannel. As discussed, the flow inside micro devices is characterized by the omnipresence of laminar flow; therefore, the flow was considered as laminar. In this study, numerical simulations were performed by assuming flow as laminar, steady, and incompressible. The detailed analysis of the mixing between the fluids was investigated by solving, continuity, mass diffusion, and three-dimensional Navier–Stokes equations. Under the considered assumptions, the equations are mathematically articulated as:

(1) $$\vec{\nabla }\;\cdot \;\vec{V\;}\;=\;0$$

(2) $$\left(\vec{V\;}\cdot \vec{\nabla }\right)\;\vec{V\;}=\;\nu \nabla^{2}\;\vec{V\;}\;+\;\frac{1}{\rho }\nabla \;p$$

(3) $$\left(\vec{V\;}\cdot \vec{\nabla }\right)c\;=\;\;D\;\nabla^{2}c$$

where, V represents the velocity, ν represents the kinematic viscosity, p represents the pressure, ρ represents the density, D represents the diffusivity constant, and the mass fraction of species of the mixing fluids is denoted by c, respectively. The fluid flow inside the micromixer was defined as single-phase multi-component fluid flow. Therefore, apart from the transport equations, one other equation must be taken into account that depicts the effect of the variation [28] of the fluid properties along the flow:

(4) $$\frac{\partial \rho_{i}}{\partial t}\;+\;\frac{\partial \left(\rho_{i}{\vec{V\;}}_{j}\right)}{\partial x_{j}}\;=\;-\frac{\partial \left[\rho_{i}\left({\vec{V\;}}_{\mathrm{ij}}-{\vec{V\;}}_{j}\right)\right]}{\partial x_{j}}\;+\;S_{i}$$

where, $\rho_{i}$ represents the fluid density at sample point i within the mixture, ${\vec{V\;}}_{j}$ represents average velocity field, ${\vec{V\;}}_{\mathrm{ij}}$ represents the average velocity of ith component within the mixture, and $S_{i}$ represents the source term. For the entire domain, $S_{i}$ must be equal to zero; therefore, when the above equation is applied and the results are summed for each of the components of the mixture, then the reduces to the following form:

(5) $$\frac{\partial \left(\rho_{i}{\vec{V\;}}_{j}\right)}{\partial x_{j}}\;=\;0$$

where the equation is equivalent to the mass continuity equation of the fluid flow. Therefore, single velocity can be used for the computational analysis. In this study, water and the combination of dye–water was inserted through inlet 1 and inlet 2. It was assumed that temperature was constant at 20 ℃. The dynamic viscosity (μ) and density (ρ) of the water were considered 8.84 × 10^−4^ kg/m s and 997 kg/m^3^, respectively [29]. The diffusion coefficient for the dye–water mixture was constant throughout the numerical simulations at 1 × 10^−11^ m^2^/s [18]. The numerical solution was executed by ANSYS CFX 15.0 [28] for principal equations. A high-density hexahedral mesh was created within the computational domain using ANSYS-ICEM CFD 15.0. The mesh density at the junction of the mixing units was kept high. The advection term was discretized using the high-resolution of the second-order approximation. The aspect ratio of cells was kept near to unity so that the numerical diffusion error was minimized and highly precise numerical results were obtained [30]. Additionally, for discretization of the convective parts, a high-resolution method [28] was used in the principal equations. Moreover, for pressure velocity pairing, the SIMPLEC algorithm [31] was used. 

For numerical simulations, diverse boundary conditions were applied at the outlet, inlets, and side walls. Normal inlet velocity was calculated on the basis of the properties of water and specified as the inlet boundary condition at both inlets. Atmospheric pressure (zero) was applied at the outlet section, and the frictionless wall was specified. The convergence criteria of numerical solution were taken in terms of the root mean square value (10^−6^) at each node of the computational domain.

## 4. Evaluation Parameter for the Performance of Micromixer

On the basis of variance of the dye mass fraction across a cross-sectional plane, the mixing efficiency was determined. The plane was taken at the end of the last mixing unit, and the mass fraction variation was calculated using following formula:

(6) $$\sigma \;=\;\sqrt{\frac{1}{N}{\left(c_{i}-{\bar{c\;}}_{m}\right)}^{2}}$$

where, σ represents the standard variation across the sample plane, dye mass fraction at the *i*^th^ sample point is denoted by c*_i_*, c_m_ represents the mass fraction (mean) on the cross-sectional plane, and the number of sample points is denoted by N. Subsequently, the following mathematical expression was used to calculate the mixing index:

(7) $$M\;=\;1\;-\;\sqrt{\frac{\sigma^{2}}{\sigma_{\max}^{2}}}$$

where, σ^2^ is the variation of the dye mass fraction, and the maximum variance value on the sample plane is denoted by the $\sigma_{\max}^{2}$. Variance in the mass fraction is inversely related to the mixing index across the sample plane. For the best performance of a micromixer, the numerical value of the mixing index should be 1.0, which corresponds to the minimum variance of dye mass fraction.

## 5. Selection of Design Constraints and Objective Functions

The primary and very fundamental process during the optimization process is to select the appropriate design constraints that influence the objective function values. A detailed parametric investigation was carried out to select the sensitive parameters for structural optimization. Three dimensionless geometric parameters: the ratio of the main channel thickness to the pitch length (H/PI), the ratio of the thickness of the diagonal channel to the pitch length (W/PI), and the ratio of the depth of the channel to the pitch length (d/PI) were chosen to optimize the proposed micromixer. The design ranges of the parameters were restricted based on a preliminary study, shown in Table 1. For numerical analysis, the LHS method was applied to finalize the (twenty-eight) design points. A commonly used mixing index (F_MI_) of the micromixer was considered for objective function values. 

## 6. Methodology of the Single-Objective Optimization

**Figure 2:**
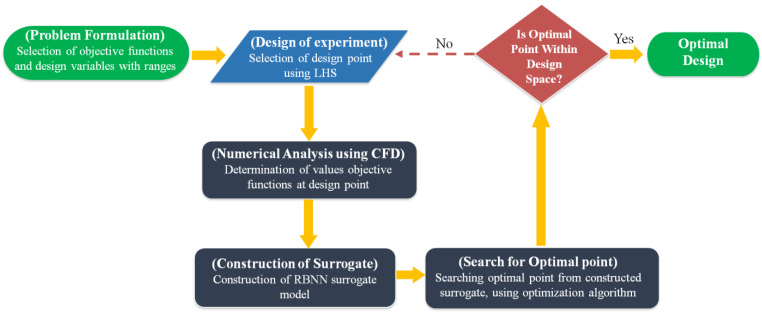
Flow process for single-objective optimization method. LHS = Latin hypercube sampling; CFD = computational fluid dynamics; RBNN = radial basis neural network.

Figure 2 demonstrates the optimization procedure on the basis of surrogate modeling. Single-objective optimization was applied for the structural optimization. The optimization study, formulated as the maximization of the objective function value, can be mathematically articulated as:

Min. F(*x*) subjected to *x*_l_ ≤ *x* ≤ *x*_u_

where, x_u_ and x_l_ represent the upper and lower limits of the design variable *x*, respectively. In this study, the optimization procedure maintained the following steps; initially effective design parameters and proper design constraints were determined through the preliminary study to enhance the performance of the micromixer. 

To formulate the surrogate model, design of experiments (DOE) was applied to select the uniformly distributed design points within the design constraint. Two types of DOE methods are found in the literature: random design and orthogonal design. In the orthogonal design technique, model parameters are self-determining and represent that the factors are not related (experimentally) and can be varied separately. There are some limitations of orthogonal designs. Firstly, they are quite unconvincing when it comes to determining the important factor. As the fundamental function is determined, the probability of reproducing the design points is high, generally termed as the collapse problem [32]. Therefore, the method is inefficient in terms of the computational time period. To conquer the problem, a random design was applied to confirm the design points for the structural optimization. In a random design, design parameter values are determined on the basis of a random process [33]. As a result, there is no chance of reproducibility of the design points. Thus, each point provides unique information about the effect of another factor on the response. Accordingly, the method is very efficient in terms of computational time period [33].

In the present work, a random design based on LHS [34] was chosen to formulate the surrogate models with which to estimate the values of the objective function. Generally, the LHS method uses an m × n matrix, where sampling points are denoted by m and design parameters are denoted by n, respectively. In an LHS matrix, every n column contains the levels 1, 2, 3 tom, arbitrarily grouped to structure a Latin hypercube. Thus, the technique generates random sampling within the data range, which confirms every segment of the design constraint. To determine the optimum points within the design constraint a (GA) genetic algorithm [35] was considered for the search algorithm. For design points, the well-known MATLAB function (i.e., lhsdesign) was used. To exaggerate the minimum distance among neighboring design points, maxmin was used [36]. The values of objective functions at selected design points (twenty-eight) were calculated numerically. 

Next, a surrogate was formulated depending on the (twenty-eight) objective function values. To determine the optimal points within the design constraint, the algorithms needed a large number of estimations of objective function. Therefore, for the sake of computational time saving, it was obligatory to formulate a surrogate model on the basis of distinct numerical analysis within the design constraints. In this study, to estimate the objective function values, the RBNN technique was used [37]. 

The RBNN is an artificial neural network (ANN) that considers the radial basis functions (RBF) as activation functions, constructed by three layers (i.e., hidden layer, input layer, and output layer), which contain linear or nonlinear neurons. Each activation function depends on the distance from the center vector to the input layer; thus, the function becomes symmetric along the center vector [38]. The hidden layer contains a set of functions of radial basis, which perform the same as activation functions [38]. The response differs within the distance between the center and the input. Furthermore, variation of coordinates was used to determine the distance between the center and the input. The radial basis model is able to reduce computational time as well as cost using its linear nature of the functions. Using the number of N basis functions of the model (linear), f (x) is mathematically expressed as:

(8) $$f\;\left(x\right)\;=\;\overset{N}{\underset{j=1}{\sum }}w_{j}y_{j}$$

where weight is represented by w_j_,and y_j_ is the basis function. Various techniques can be applied to select the functions. The function can be classified as nonlinear or linear. For the linear model, the parameters, including the basis function, are stable throughout the iteration progression. On the other hand, if the basis function differs through the iteration, it calls for a nonlinear model. The iteration procedure, corresponding to a search for the best plane within the multidimensional space, offers a suitable match to the learning data. The parameters of the surrogate model are termed as a user-defined error goal (EG) and the spread constant (SC) [38]. The finding of an appropriate value of EG and SC is very crucial. A large value of the EG will affect the precision of the model, whereas a small value of the EG will construct a model larger than the training experience of the network. Furthermore, estimation of the proper values of the SC is very crucial; if the SC value is large, the neuron may not react identically at each input; whereas for a small value of SC, the network would be highly sensitive. The appropriate EG value is resolute from the acceptable error of the mean input responses. The proposed modified RBNN function, called “newrb”, accessible in MATLAB, was used in this study [39]. 

## 7. Results and Discussions 

**Figure 3:**
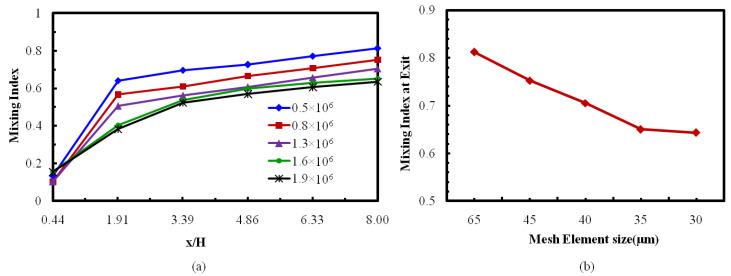
Grid dependency examination for reference design. (**a**) Mixing index with the channel length. (**b**) Mixing index at (x/H = 8.0) exit.

In any computational study to ensure a high-quality grid system, it is a predominant criterion to diminish the numerical inaccuracies generated during the discretization process. In this study a tetrahedral grid was built for the computational domain. To establish an optimum number of nodes as well as mesh size, an investigation of grid-dependency was executed. A grid system containing a number of nodes (five) starting from 0.5 × 10^6^ to 1.9 × 10^6^ were tested for mixing index evaluation down to the microchannel length at Reynolds numbers (Re = 40), as shown in Figure 3. To evaluate the mixing indices, perpendicular planes at six different positions along the axial direction were considered (Figure 3a). An almost identical development of the mixing performance is depicted for nodes 1.6 × 10^6^ and 1.6 × 10^6^, Re = 40. Additionally, tiny variation was found in the mixing development (0.27%) at the exit of the micromixer between the two grid systems, represented by the mesh element sizes 3.5 μm and 3.0 μm, respectively (Figure 3b). Therefore, from the tested results, the grid system representing nodes 1.6 × 10^6^ (i.e., mesh element size 3.5 μm) was considered to be the most favorable grid system. 

**Figure 4:**
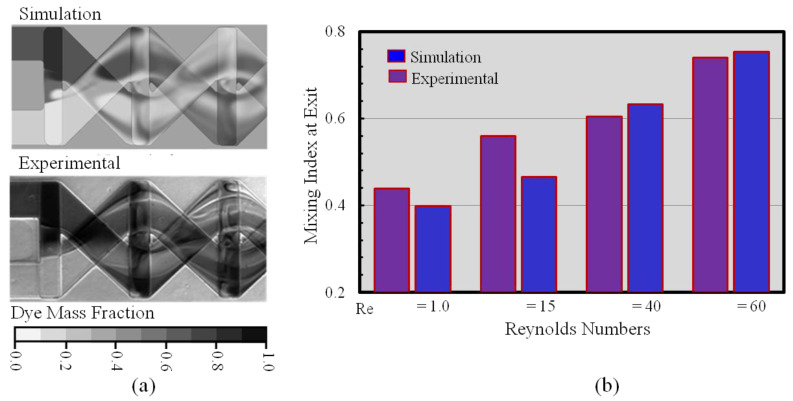
Confirmation of proposed model (numerical) evaluated by experimental findings [27]: (**a**) qualitative evaluation (Re = 60) and (**b**) quantitative evaluation at Re = 1, 15, 40, and 60.

The numerical model was validated qualitatively and quantitatively with experimental findings [27], as demonstrated in Figure 4. The induced uncertainties during the experimental process were wall unevenness and dimensional variation (±5 µm) in fabrication. The phenomena play a vital role in the variations of mixing performance (Figure 4b). The numerical scheme was validated quantitatively and qualitatively with experimental results, shown in Figure 4a. The visual photograph of the dye mass fraction distribution of fluid mixing was confirmed with the numerical result on the x–y plane situated at the center of the top and bottom channel depth (Figure 4a). The following graph (4b) represents mixing indices at Re = 1, 15, 40, and 60, which were quantitatively evaluates with experimental data [27]. Numerical prediction values of the mixing indices were marginally varied with experimental data throughout the Reynolds number range. The variation between the experimental and numerical results occurred due to the microchannel fabrication procedure, including dimension variations (±5 μm), wall roughness, and experimental uncertainties such as focusing and evaluating the experimental images. Nevertheless, the quantitative and qualitative evaluation between the experimental and numerical results demonstrates satisfactory agreement.

**Figure 5:**
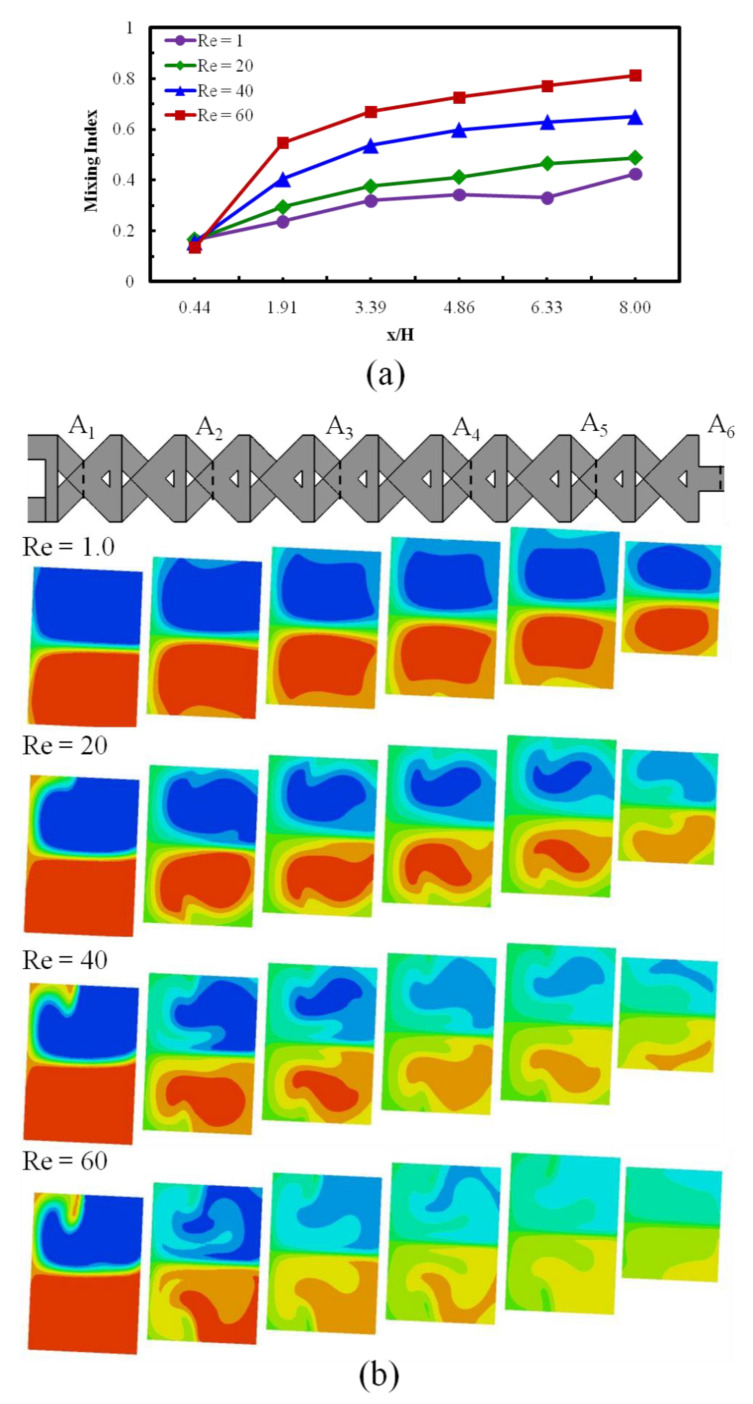
Mixing index development for reference design micromixer at different Reynolds numbers (Re = 1.0, 20, 40, and 60). (**a**) Improvement of mixing index along the microchannel. (**b**) Captured images of mass fraction distributions (dye) on cross-sectional planes.

Consequences of the Reynolds numbers on mixing performance were examined both quantitatively and qualitatively through a numerical procedure. To demonstrate the mixing developments, the mass fraction variations were captured on six cross-sectional planes (A_1_–A_6_) at the crossing nodes, shown in Figure 5a. At Re = 1.0 (Figure 5b), the interfacial area of the miscible sample is practically visible, has relatively fewer distortions, and is straight at each cross-sectional plane. Figure 5b shows two symmetric (at top and bottom) transverse fluid flow patterns. The figure demonstrates that the interfacial surface of the fluid becomes progressively wider as the flow proceeds and also with the Reynold numbers. The developments of mixing indices (Figure 5b) to the down way of the micromixer at four various Reynolds numbers (Re = 1, 20, 40, and 60) is presented. Six cross-sectional planes were selected for the estimation of the mixing index at the middle of the crossing structures. Figure 5b shows that increasing phenomena of mixing performance significantly vary with the Reynolds numbers.

**Figure 6:**
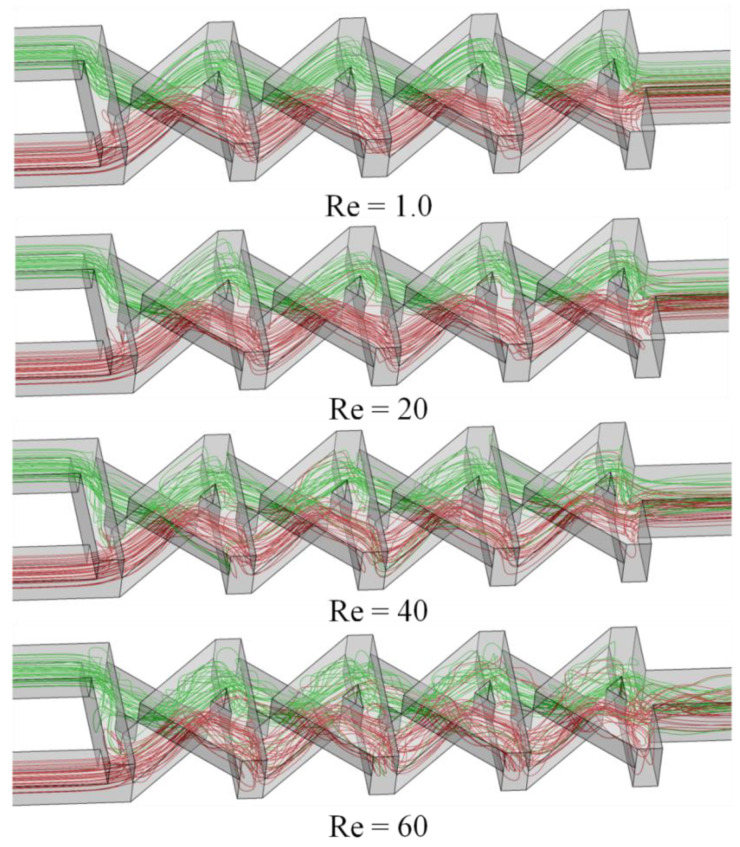
Streamlines originated from different inlets in the reference design micromixer for Re = 1.0, 20, 40, and 60.

Figure 6 illustrates the 3D streamlines of fluids, indicated with two different colors, originated from both inlets at Re = 1.0, 20, 40, and 60, which are plotted to scrutinize the fluid flow structure that enhances the mixing performance. Initially, the fluids start mixing at the middle of the first vertical section and enter to the main channel. Due to the 3D channel structure, the streams of fluid maintain their flow path after impact (Figure 1b). Thereafter, the fluid streams recombine at the first crossing node (A1 section). A fraction of swapping of streamlines happens at the crossing nodes, which produces an enlarging and shrinking of the fluids interface, and thus promotes chaotic advection. Therefore, the streams of fluids are gradually divided into many layers during a progression of ten mixing segments. The flow mechanism enlarges the interfacial section of fluids and diminishes the length of diffusion across the fluids’ layers, and thus assists quicker diffusion and fast mixing. These phenomena increase with the Reynolds numbers, and thus enhance the mixing performance.

**Figure 7:**
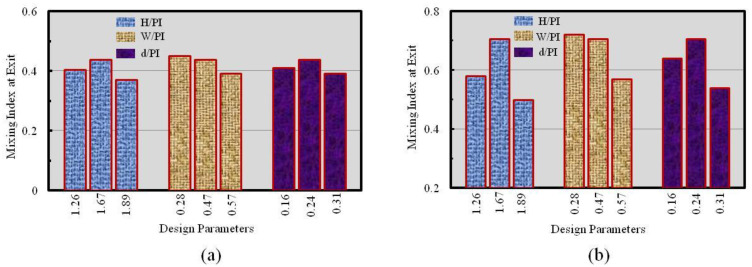
Effect of the geometrical parameters on the values of mixing performance with three dimensionless parameters at (**a**) Re = 1.0 and (**b**) Re = 40.

Outcome of the structural design parameters (i.e., H/PI, W/PI, and d/PI) on the behavior of the mixing performance at Re = 1.0 and Re = 40 was performed as shown in Figure 7; three dimensionless parameters (i.e., H/PI, W/PI, and d/PI) listed in Table 1 were considered for numerical investigations. A number of mixing segments (ten) were kept constant; thus, the down way length of the micromixer remained the same during the investigations. At Re = 1.0, the mixing index was not significantly varied (Figure 7a) with the geometric parameters; thus, it is concluded that for this Reynolds number the mixing index generally depended on the molecular transmission of fluids rather than the geometric parameters. Mixing performance at Re = 40 (Figure 7b) is slightly varied with the value of W/PI. Parametric findings representing the variation of mixing index values were very much reactive to H/PI (42% deviation for 1.26 < H/PI < 1.89) than to d/PI (31% deviation for 0.16 < d/PI < 0.31) and W/PI (25% deviation for 0.28 < W/PI < 0.57). For predetermined down way length of the microchannel, the channel width (W) was proportionally varied along the values of channel width (H); thus, intensity of sample fluid velocity diminished as the H/PI value increased. Hence, lesser velocity corresponded with high residual duration of the fluid flow within the microchannel and weaker inertia force of fluids. The maximum mixing index value 0.72 was found at W/PI = 0.28 (minimum value), where the best matching of the fluids interfacial area and residential time was obtained. Thus, the higher value of W/PI caused a lower mixing index value at the exit.

**Figure 8:**
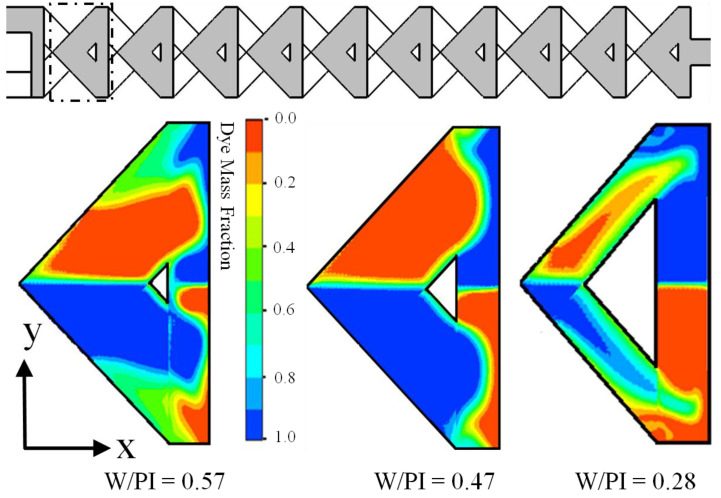
Dye mass fraction distributions at the middle of the top channel (designated by the dotted lines) of the first mixing unit for different values of W/PI = 0.57, 0.47, and 0.28 for reference design micromixers at Re = 40.

For qualitative evaluations of mixing performance values, mass fraction distributions were plotted (on x–y planes) at Re = 40, shown in Figure 8. The dye mass fraction distributions are plotted at the center of the top layer microchannel (as indicated by the dotted rectangular box) of the first mixing unit. With the increase of the W/PI values, the interface of the fluids becomes wider; additionally, mixing performance increases (Figure 7b). The inertia force of fluids and the values of W/PI are inversely proportional. The stronger inertia force of fluid (with lower values of W/PI) enhances the chaotic advection within the micromixer, and thus the mixing performance at the exit improves significantly. 

In this study, the well-known surrogate models, RBNN, were usedto achieve the optimized micromixer.Table 2 shows the design variables values (H/PI = 1.73, W/PI = 0.42, and d/PI = 0.18) and objective function (MI = 0.86) for an optimum micromixer using the surrogate model. Predicted design variable values of the optimum micromixer were compared with the reference micromixer. Table 2 also represents the objective function values of the reference design (0.65) and predicted optimal design (0.86), representing a 32% relative increase in the mixing index through surrogate-based optimization. Considering the reference design, the optimum design was found at the lower value of d/PI (0.18). The calculated objective function value using the Navier–Stokes equation also compared with the predicted values, 0.86 and 0.81, respectively. This comparison signifies that the surrogate based optimization technique shows 6.2% deviation of objective function value from the optimum point. 

**Figure 9:**
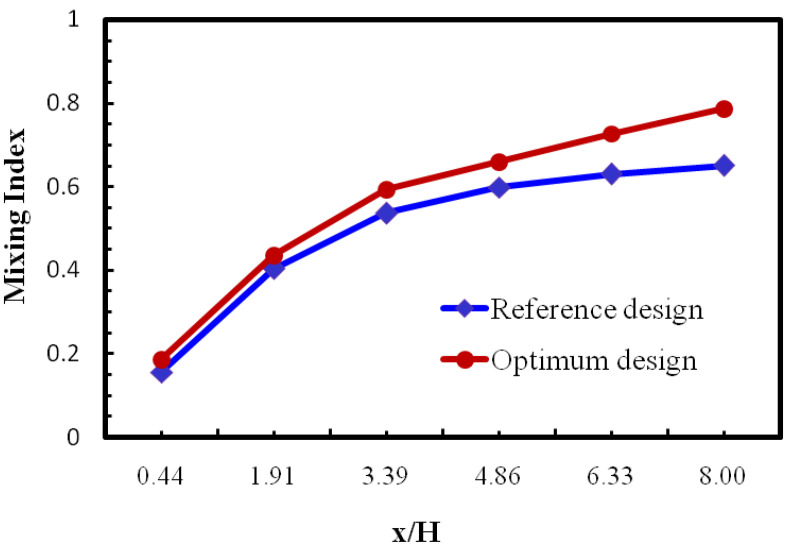
Improvement of objective function values along the channel length for reference design and optimum designs at Re = 15.

The developments of objective function (MI) value along the down way length of the micromixer for optimized geometry and reference design are represented at Reynolds number 40. Developing rates of the mixing index increase for both designs along the channel length, depicted in Figure 9. Figure 9 also represents the optimum design as having better mixing performance throughout the microchannel length, and the value of the mixing index-optimized design micromixer (x/H = 8.0) as having 1.4 times higher performance compared to the reference design. 

**Figure 10:**
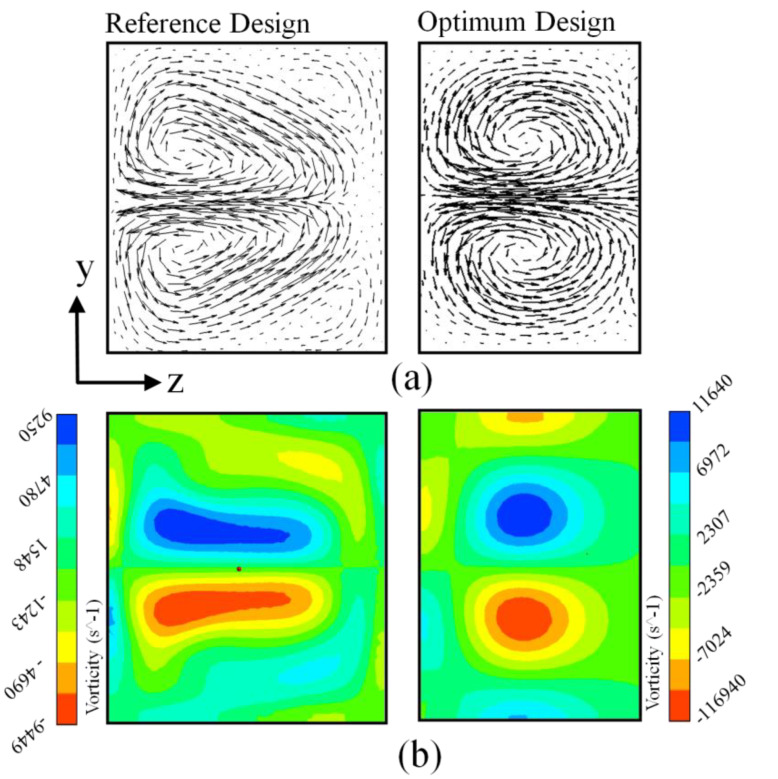
Qualitative comparison of reference and optimum designs at Re = 40. (**a**) Velocity vectors plot and (**b**) local vorticity distributions plot, on y–z planes (at x/H = 8.0).

**Figure 11:**
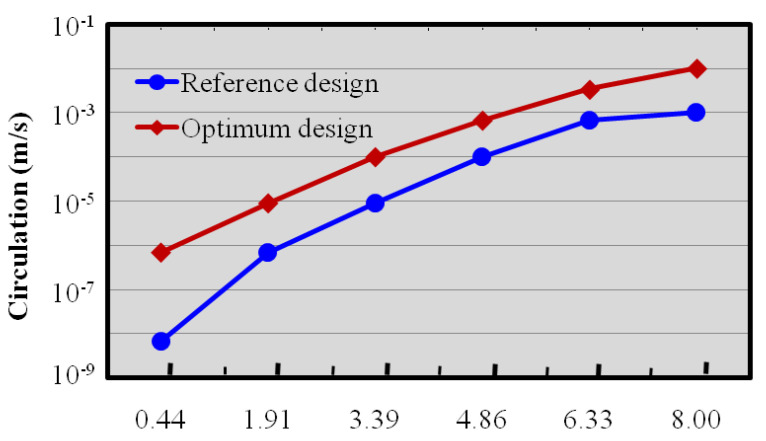
Normalized circulation distributions for reference design and optimum design at Re = 40.

**Figure 12:**
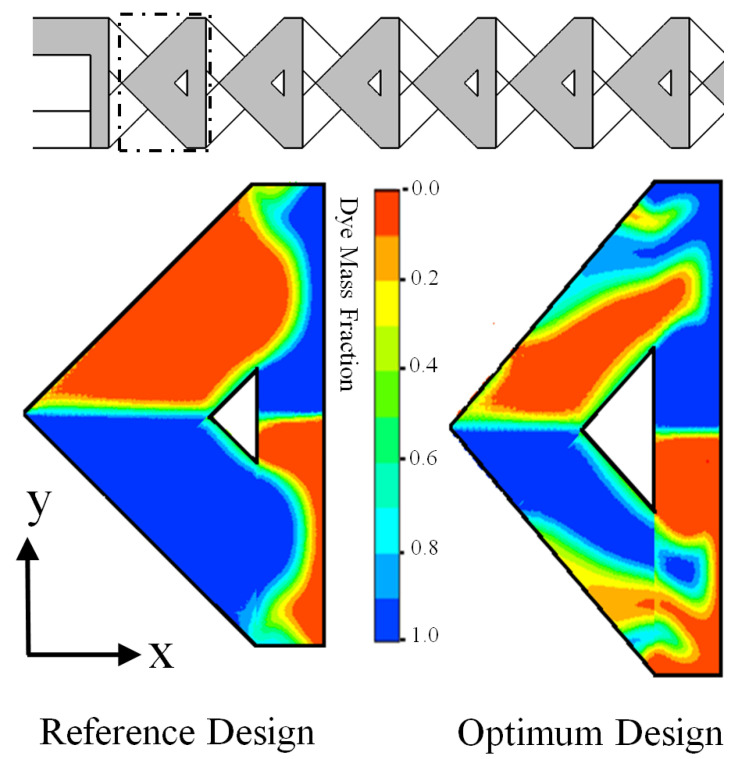
Mass fraction distributions of dye at the middle of the top channel (designated by the dotted lines) of first mixing unit for reference design and the optimum design micromixers at Re = 40.

Figure 10a,b shows the velocity vectors plot and local vorticity variations on y–z planes of the reference and optimum designs, respectively. The cross-sectional plane was plotted (x/H = 8.0) at the end of the last mixing segment. A pair of counter-rotating vortices was observed in both cross-sectional planes. The optimized micromixer visualized a pair of small round-shaped (counter-rotating) vortices that filled the entire plane, while in the reference micromixer, two oval-shaped counter-rotating vortices shifted to the side wall; thus, velocity vectors became relatively weaker. On the other hand, the velocity vector for the optimum design micromixer indicated a strong transverse flow pattern, and velocity vectors were consistently spread all through the cross-sectional plane. The strongest transverse flow phenomena (Figure 10a) produced the difference in mixing performance for the optimized design micromixer. Figure 10b shows local vorticity distributions, plotted on the y–z plane for the reference and optimum design. The vorticity was designed by following formula:

(9) $$\omega_{x}\;=\;\left(\frac{\partial v_{z}}{\partial y}\;-\;\frac{\partial v_{y}}{\partial z}\right)$$

where, $\omega_{x}$ represents vorticity along the x-direction, and w and v are the velocity components along z and y directions. As the vorticity plot signifies, as compared to the reference design, the strength of the vorticity is augmented in the optimum design and creates the potential difference in mixing performance at the exit. Normalized circulation development along the reference design and optimum design micromixers is represented in Figure 11. Circulation values signify the potentiality of vertical movement on the plotted plane, as shown in Figure 10b. The circulation (Ω_x_) is articulated by incorporating the streamwise vorticity on y–z planes, mathematically represented as:

(10) $$\Omega_{x}\;=\int_{A_{\mathrm{yz}-\mathrm{plane}}}\left(\frac{\partial v_{z}}{\partial y}-\frac{\partial v_{y}}{\partial z}\right)\mathrm{dydz}$$

where, v_y_ represents the y direction velocity components and v_z_ represents the z direction velocity component. Along the channel length, the value of the circulation increases in both micromixers. Figure 11 illustrates that, as compared to the reference design, the optimized design has a higher circulation value, which enhances mixing performance. The dye mass fraction distributions at the middle of the top channel (designated by the dotted lines) of first mixing unit were plotted for the reference design and the optimum design micromixers, as shown in Figure 12. It is observed that the enhancement of the secondary flow induced in the optimized micromixer was superior compared to the reference design micromixer. Interface of sample fluids in the optimum design was wide and, accordingly, a quicker improvement of mixing performance along the microchannel length is observed (Figure 9). The result confirms that, for the optimum design micromixer, mixing performance is around 25% higher than the reference design micromixer.

## 8. Conclusions

Geometric optimization of a proposed two-layer crossing micromixer was carried out using 3D Navier–Stokes formulas. To estimate the objective function, the well-known RBNN model was used. The optimization study was performed with three design variables; viz., the ratio of the main channel thickness to the pitch length (H/PI), the ratio of the thickness of the diagonal channel to the pitch length (W/PI), and the ratio of the depth of the channel to the pitch length (d/PI). The mixing index for the micromixer (F_MI_) was considered as an objective function to find the most efficient design.

By this study, one can conclude the following: For Reynolds numbers ≤1.0, the objective function was not significantly varied with the geometric parameters. The parametric study representing the objective function values was very much more sensitive to H/PI than to d/PI and W/PI. The maximum mixing index value of 0.72 was found at lowest value of W/PI, where the best matching of the fluids interfacial area and residential time was obtained. Surrogate-based optimization results represented the design variables (H/PI = 1.73, W/PI = 0.42 and d/PI = 0.18) and the objective function (MI = 0.86) for the optimum micromixer. The objective function values of the reference design and predicted optimal design were 0.65 and 0.86, respectively, which confirms a 32% relative increase in the objective function through the surrogate-based optimization procedure. The calculated objective function value using Navier–Stokes equation was compared with the predicted value. The RBNN model represented a 6.2% relative deviation of objective function value from the optimum point. The study represents that the single-objective optimization procedure is favorable for the improvement of micromixer performance. The optimum micromixers could be incorporated into a micro-total analysis system and lab-on-chip (LOC) systems to facilitate the study of reaction kinetics, dilution of fluid samples, and enhancement of reaction selectivity.

## Figures and Tables

**Table 1:** Design parameters with their limits.

Design Variables	Ratio of The Main Channel Thickness to the Pitch Length (H/Pi)	Ratio of The Thickness of The Diagonal Channel to the Pitch Length (W/Pi)	Ratio of The Depth of The Channel to the Pitch Length (D/Pi)
Lower Limit	1.26	0.28	0.16
Upper Limit	1.89	0.57	0.31
Reference Design	1.67	0.47	0.24

**Table 2:** Comparison of the objective function (MI)-oriented optimum geometry with the reference geometry.

Design Variables	H/PI	W/PI	d/PI	Objective Function (MI)	
				Predation by RBNN	Calculation by Navier–Stokes Analysis
Reference Design	1.67	0.47	0.24	-	0.65
Optimum Design	1.73	0.42	0.18	0.86	0.81
